# Supplementary material for: Surfactin production is not essential for pellicle and root-associated biofilm development of Bacillus subtilis
Source: Biofilm. 2020 Mar 20;2:100021. doi: 10.1016/j.bioflm.2020.100021 (PMC7798449; doi:10.1016/j.bioflm.2020.100021)
Supplement: Multimedia component 1 [file mmc1.docx]

**Table S1. Strains used in this study**

| **B. subtilis strains** | **Characteristics** | **Reference** |
| --- | --- | --- |
| NCIB3610 | WT/Undomesticated strain | Lab stock |
| SSB46 | 3610 *srfAA::erm* | [28] |
| MT529 | 3610 *srfAA::erm* (new transduction in 3610 background) | This study |
| MT476 | 3610 *srfAA::kan* | This study |
| PB174 | *amyE::P_tapA_-lacZ* | Lab stock |
| MT644 | *sfp::erm* | BKE03569 [46] in 3610 |
| MT619 | *srfAA::kan amyE::P_tapA_-lacZ* (Spec^R^) | This study |
| MT650 | *sfp::erm amyE::P_tapA_-lacZ* (Spec^R^) | This study |
| MT607 | *amyE::P_eps_-lacZ* (Chl^R^) | Lab stock |
| MT613 | *srfAA::kan amyE::P_eps_-lacZ* (Chl^R^) | This study |
| MT651 | *sfp::erm amyE::P_eps_-lacZ* (Chl^R^) | This study |
| CA018 | *amyE::P_tapA_-yfp* (Spec^R^) | [47] |
| MT649 | *srfAA::kan amyE::P_tapA_-yfp* (Spec^R^) | This study |
| MB8_B1 | *B. subtilis* soil isolate from sample site 55.843861, 12.424770 | This study |
| MB9_B1 | *B. subtilis* soil isolate from sample site 55.843861, 12.424770 | This study |
| P5_B1 | *B. subtilis* soil isolate from sample site 55.788800, 12.558300 | This study |
| P8_B1 | *B. subtilis* soil isolate from sample site 55.795200, 12.580600 | This study |
| P9_B1 | *B. subtilis* soil isolate from sample site 55.791200, 12.575100 | This study |
| 75 | *B. subtilis* soil isolate from sample site 50.725876, 10.916218 | This study |
| DTUB27 | MB8_B1 *amyE*::P_hyperspank_-*gfp* (Chl^R^) | This study |
| DTUB30 | MB9_B1 *amyE*::P_hyperspank_-*gfp* (Chl^R^) | This study |
| DTUB38 | P5_B1 *amyE*::P_hyperspank_-*gfp* (Chl^R^) | This study |
| DTUB40 | P8_B1 *amyE*::P_hyperspank_-*gfp* (Chl^R^) | This study |
| DTUB43 | P9_B1 *amyE*::P_hyperspank_-*gfp* (Chl^R^) | This study |
| TB731 | 75 *amyE*::P_hyperspank_-*gfp* (Chl^R^) | This study |
| DS1122 | 3610 *srfAC*::tn*10* (Spec^R^) | [32] |
| DTUB68 | MB8_B1 *srfAC*::tn*10* (Spec^R^) | This study |
| DTUB71 | MB9_B1 *srfAC*::tn*10* (Spec^R^) | This study |
| DTUB79 | P5_B1 *srfAC*::tn*10* (Spec^R^) | This study |
| DTUB80 | P8_B1 *srfAC*::tn*10* (Spec^R^) | This study |
| DTUB82 | P9_B1 *srfAC*::tn*10* (Spec^R^) | This study |
| DTUB89 | 75 *srfAC*::tn*10* (Spec^R^) | This study |
| DTUB146 | MB8_B1 *srfAC*::tn*10* (Spec^R^); *amyE*::P_hyperspank_-*gfp* (Chl^R^) | This study |
| DTUB147 | MB9_B1 *srfAC*::tn*10* (Spec^R^); *amyE*::P_hyperspank_-*gfp* (Chl^R^) | This study |
| DTUB148 | P5_B1 *srfAC*::tn*10* (Spec^R^);*amyE*::P_hyperspank_-*gfp* (Chl^R^) | This study |
| DTUB149 | P8_B1 *srfAC*::tn*10* (Spec^R^); *amyE*::P_hyperspank_-*gfp* (Chl^R^) | This study |
| DTUB150 | P9_B1 *srfAC*::tn*10* (Spec^R^); *amyE*::P_hyperspank_-*gfp* (Chl^R^) | This study |
| DTUB145 | 75 *srfAC*::tn*10* (Spec^R^); *amyE*::P_hyperspank_-*gfp* (Chl^R^) | This study |

[46] Koo B-M, Kritikos G, Farelli JD, Todor H, Tong K, Kimsey H, et al. Construction and analysis of two genome-scale deletion libraries for *Bacillus subtilis*. Cell Syst 2017;0:1–15. doi:10.1016/j.cels.2016.12.013.

[47] Vlamakis H, Aguilar C, Losick R, Kolter R. Control of cell fate by the formation of an architecturally complex bacterial community. Genes Dev 2008;22:945–53. doi:10.1101/gad.1645008.
