# Supplementary material for: Surfactin production is not essential for pellicle and root-associated biofilm development of Bacillus subtilis
Source: Biofilm. 2020 Mar 20;2:100021. doi: 10.1016/j.bioflm.2020.100021 (PMC7798449; doi:10.1016/j.bioflm.2020.100021)
Supplement: Multimedia component 2 [file mmc2.docx]

**Fig S1**. Pellicle formation with deletion mutants identified as containing mutations in strain SSB46. Pellicles were formed in MSgg for 24 h at 30 ºC. Pictures are representative of biological duplicates.

**Fig S2**. Impact of NRPs mutations on colony complexity. Top-down view of colonies incubated for 72 h at 30 °C on solid MSgg or MSNc + pectin. Results are representative of at least two experiments. Scale bars are 5 mm.

**Fig S3**. Extracted ion chromatograms (m/z 1000-2000) showing the presence of surfactin produced by the newly isolated *B. subtilis* strains grown on MSgg agar medium and the lack of surfactin production in the *srfAC* derivates. The chromatograms of the MSgg medium and the surfactin standard are shown below. Surfactins, iturins and fengycins are all in the m/z range 1000–2000 that can be detected by ESI–MS [45,46].
